# Lathyrol reduces the RCC invasion and incidence of EMT via affecting the expression of AR and SPHK2 in RCC mice

**DOI:** 10.1007/s12672-024-01130-w

**Published:** 2024-07-04

**Authors:** Shengyou Song, Lunwei Tai, Yuqi Xu, Junling Jiang, Lei Zhou, Junfeng Zhao

**Affiliations:** 1 Department of Urology, The Second Affiliated Hospital of Henan University of Traditional Chinese Medicine, Zhengzhou, 450002 Henan China; 2 Department of Urology, The Second Affiliated Hospital of Henan University of Traditional Chinese Medicine, Dongfeng Road 6#, Zhengzhou, 450002 Henan China

**Keywords:** Lathyrol, Renal cell carcinoma, Androgen receptor, Epithelial-mesenchymal transition, Invasion

## Abstract

**Objective:**

To investigate the effects of Lathyrol on the expression of androgen receptor (AR) and sphingosine kinase 2 (SPHK2) in renal cell carcinoma (RCC) mice and to further explore the mechanism by which Lathyrol inhibits the invasion and incidence of epithelial-mesenchymal transition (EMT).

**Methods:**

An RCC xenograft mouse model was constructed, and the mice were randomly divided into a model group, an experiment group and a negative control group. The experiment group was intragastrically gavaged with Lathyrol solution (20 mg/kg), the model group was intragastrically gavaged with 0.9% NaCl (same volume as that used in the experiment group), and the negative control group was injected intraperitoneally with 2 mg/kg cisplatin aqueous solution. Changes in the body weight and tumor volume of the mice were recorded. Western blot (WB) was used to assess the protein expression levels of AR, p-AR, CYP17A1, PARP1, E-cadherin, N-cadherin, vimentin, α-SMA, β-catenin, and ZO-1. Protein expression levels of SPHK2, metal matrix protease 2 (MMP2), MMP9 and urokinase-type plasminogen activator (uPA) in tumor tissues were assessed by immunohistochemistry (IHC). AR expression in tumor tissues was assessed after immunofluorescence (IF) staining.

**Results:**

After 14 days of drug administration, compared with that in the model group, the tumor volumes in the negative control and experiment groups were lower; the difference in tumor volume among the model, control and experiment groups was statistically significant (*P* < 0.05). The differences in body weight among the three groups were not statistically significant (*P* > 0.05). In the model group, the protein expression levels of AR, p-AR, CYP17A1, SPHK2, and PARP1 were relatively increased, the protein expression levels of E-cadherin and ZO-1 were relatively reduced (*P* < 0.05), and the protein expression levels of N-cadherin, β-catenin, vimentin, and α-SMA were relatively increased (*P* < 0.05). In the negative control and experiment groups, the protein expression levels of AR, p-AR, CYP17A1, SPHK2, and PARP1 were relatively decreased (*P* < 0.05), the protein expression levels of E-cadherin and ZO-1 were relatively increased (P < 0.05), and the protein expression levels of N-cadherin, β-catenin, vimentin and α-SMA were relatively decreased (*P* < 0.05).

**Conclusion:**

Lathyrol and cisplatin inhibit the proliferation of RCC xenografts, reduce the protein expression levels of AR, CYP17A1, SPHK2, PARP1, E-cadherin, and ZO-1 in tumor tissues (*P* < 0.05), and promote the protein expression levels of N-cadherin, β-catenin, vimentin and α-SMA (*P* < 0.05). Therefore, Lathyrol reduces RCC invasion and EMT by affecting the expression of AR and SPHK2 in RCC mice.

**Supplementary Information:**

The online version contains supplementary material available at 10.1007/s12672-024-01130-w.

## Introduction

Renal cell carcinoma (RCC) is a common urinary tract tumor, accounting for approximately 3% of adult malignancies in terms of incidence [1], and ranks second among urological tumors; in mice, RCC is the most lethal tumor of the urinary system [2, 3].

Androgen receptor (AR) has been a key therapeutic target in cancers in recent years. AR is not only expressed in urological tumors but can also be a key target in the treatment of other systemic tumors [4]. AR is expressed in the majority of primary and metastatic prostate cancer (Pca), and its activation and expression are closely related to the occurrence and development of primary and metastatic Pca and the regulation of tumor proliferation, invasion and metastasis [5]. Sphingosine kinase 2 (SPHK2) is a multifunctional lipid kinase that regulates various molecular mechanisms. Through in-depth research, experts have established that SPHK-2 plays a key role in the pathogenesis and development of cancers. SPHK-2 activation promotes the proliferation of cancer cells and the progression of inflammation, and SPHK-2 is a key target in the treatment of cancers [6]. However, there are relatively few studies of AR and SPHK-2 in RCC, and RCC is not sensitive to radiotherapy and chemotherapy [7]. Given the poor prognosis and low five-year survival rate of RCC patients [8], novel diagnostic and treatment measures and directions are urgently needed. Immune-targeted therapy has gradually become a new research direction for the diagnosis and treatment of RCC patients [9]. Compared with classic clinical drugs, traditional Chinese medicines (TCMs) have higher economic benefits and fewer side effects. TCMs can not only directly kill tumor cells to inhibit cancer but also play an auxiliary role in anti-cancer treatment by promoting patient immunity and regulating the physical condition of patients [10]. In recent years, the combination of TCMs and clinical anti-cancer drugs to kill cancer cells and improve patients’ post-operative recovery and prognosis has become a new trend in clinical cancer diagnosis and treatment [11].

Currently, there are no reports on whether Lathyrol inhibits RCC invasion and epithelial-mesenchymal transition (EMT) through the inhibition of AR and SPHK2 expression. Therefore, on the basis of previous studies, the objectives of this study were to investigate whether Lathyrol affects RCC invasion and EMT in RCC mice by affecting the expression of AR and SPHK2 and to further elucidate the anticancer effect of Lathyrol.

## Materials and methods

### Materials

#### Main materials and reagents

The following materials and reagents were used: Lathyrol (WeiKeQi Biotechnology Company, wkq-00424); cisplatin injection (Jiangsu Hansoh Pharmaceutical Group Co., Ltd., Drug Administration Code (DAC): H20010743); complete 1640 medium (Procell Biotechnology Co., Ltd.); phosphate-buffered saline (PBS) buffer (Biosharp Company); trypsin-ethylenediaminetetraacetic acid (EDTA) (0.25%) digestion solution, enhanced chemiluminescence (ECL) developer, cell culture grade dimethyl sulfoxide (DMSO), tissue and cell radioimmunoprecipitation assay (RIPA) lysis buffer, phenylmethylsulfonyl fluoride (PMSF), goat blocking serum, hydrogen peroxide (H_2_O_2_), Mayer’s hematoxylin staining solution and neutral gum for immunohistochemistry (IHC), bicinchoninic acid (BCA) protein quantification kit, horseradish peroxidase (HRP)-labeled goat anti-rabbit secondary antibody (Beyotime Biotechnology Company); rabbit polyclonal anti-glyceraldehyde-3-phosphate dehydrogenase (GAPDH; internal reference) antibody (Wuhan Proteintech Company); rabbit polyclonal anti-CYP17A1 antibody (Huabio Biotechnology); rabbit polyclonal anti-PARP1 antibody (CST Company); anti-p-AR antibody (Absin Biotechnology, phosphorylation site pS650); Universal anti-mouse/rabbit IHC kit and protein marker (10-180KD), anti-metal matrix protease 2 (MMP2) antibody, anti-MMP9 antibody, anti-urokinase-type plasminogen activator (uPA) antibody, and anti-SPHK2 antibody (Wuhan Proteintech Company); rabbit polyclonal anti-AR antibody, anti-E-cadherin antibody, anti-N-cadherin antibody, anti-vimentin antibody, anti-α-SMA antibody, anti-β-catenin antibody and anti-ZO-1 antibody (Wanlei Biotechnology Company); EDTA, citrate solution for antigen retrieval, 4',6-diamidino-2-phenylindole (DAPI) nuclear stain, and trichostatin A (TSA) immunofluorescence (IF) reagent; and anti-fluorescence quenching mounting medium (Wuhan Baixindu Company).

#### Cells and animals

Mouse RCC cells were obtained from Shanghai Zhong Qiao Xin Zhou Biotechnology Co., Ltd., ZQ0996. BALB/c male SPF mice (SPF (Beijing) Biotechnology Co., Ltd., license: SCXK (Beijing) 2019–0010), 1.5-2-months-old and weighing 20 ± 2 g, were reared in the Laboratory Animal Centre of Henan Provincial Hospital of Traditional Chinese Medicine at 25℃ in individually ventilated cages (IVCs) with food and water ad libitum.

#### Ethics statement

This study was approved by the Central Laboratory Ethics Committee of the Second Affiliated Hospital of Henan Provincial Hospital of Traditional Chinese Medicine (acceptance number: SL-HNSZYY-2023–002; approval number: PZ-HNSZYY-2023–002). All methods were carried out in accordance with relevant guidelines and regulations. The study conforms to the ARIVE guidelines and should be carried out in accordance with the 1964 Declaration of Helsinki and its later amendments or comparable ethical standards, the U.K. Animals (Scientific Procedures) Act, 1986 and associated guidelines, EU Directive 2010/63/EU for animal experiments, or the National Institutes of Health guide for the care and use of Laboratory animals (NIH Publications No. 8023, revised 1978). The Ethics committee allows tumor burden of 2 cm or about 10% of the body weight in mice. The maximum tumor burden recorded in the current study did not exceed 10% of the body weight.

### Methods

#### Cell culture

Renca cancer cells were routinely cultured in complete RPMI-1640 medium containing 10% FBS and 1% penicillin and streptomycin in a cell incubator at 37 °C in 5% CO_2_ and 95% humidity. After 1–2 days, the medium was changed. When reaching a confluence of 70%-80%, the cells were digested in 0.25% trypsin + 0.02% EDTA digestion solution and passaged. After proliferating into the logarithmic growth phase, the cells were collected by centrifugation and subjected to subsequent experiments.

#### Construction of the RCC mouse model

After the cells were collected by centrifugation, the initial cell suspension was prepared in complete RPMI-1640 medium containing 10% FBS and adjusted to 1 × 10^7^ cells/ml. Depilatory cream was used to remove the hair and expose the skin of the axilla (right forelimb). Renca cell suspension (0.15 mL) was injected into the subcutaneous tissue of each mouse in the axilla (right forelimb), and the mice were reared in SPF-grade IVCs. The mice were allowed to eat and drink normally. The entire animal husbandry process and all experimental operations in this study complied with relevant experimental management requirements and ethical requirements for experimental animal welfare.

#### Grouping and treatment

Using the SPSS random number generator, the mice were randomly divided into a model group, a Lathyrol experiment group, and a negative control group, with 5 mice/group after excluding mice that died from tumor ulceration, infection, and fighting. After the establishment of the RCC mouse model, tumor growth was closely observed. It took approximately 14 days for tumors to grow to 5 mm^3^. The model group was gavaged with normal saline, the Lathyrol experiment group was gavaged with 0.02 g/kg Lathyrol solution once a day, and the negative control group was intraperitoneally injected with 2 mg/kg cisplatin on days 0, 3, 7, 10, and 14, twice a week. After 14 days of treatment, the mice were anesthetized, and blood was collected from the eyeball; after centrifuging the blood at 3,000 rpm for 15 min, the supernatant was stored in a -80 °C freezer. The xenografted tumor mass was isolated, and impurities and contaminated blood were washed away with PBS. The spleen, kidneys and other organs of the mice in each group were weighed.

#### Measurement of tumor volume and plot of growth curves

After tumor cell inoculation, tumor formation in the mice in each group was observed every day. After the tumor volume grew to approximately 5 mm^3^ and the volume stabilized, the body weight of the mice was measured every day, and the largest diameter (a), minor axis (b) and height (c) of the xenografted tumor mass were measured with a Vernier caliper. The tumor volume was calculated with the following formula: V = (a × b × c)/2, in mm^3^. The average weight of the mice and average tumor volume in each group were calculated, and time-xenograft tumor growth curves were plotted by GraphPad Prism.

#### IF staining for the expression and location of AR in tumor tissues

Staining was performed according to the procedures of the IF staining kit. Tumor samples were fixed in 4% paraformaldehyde solution for 72 h, dehydrated, embedded in paraffin, sectioned (5-μm thick), and dehydrated in gradient xylene and ethanol. Tissue sections were placed in a container filled with citric acid-based retrieval solution (0.01 mol/L). The container was placed in a microwave oven for 5 min under high heat and then placed at room temperature for 10 min; this process was repeated 3 times. After the retrieval solution cooled to room temperature, the container was rinsed 3 times with PBS (3–5 min per wash), and 3% H_2_O_2_ was added dropwise to the sections to block endogenous peroxidase. Goat serum working solution was added dropwise, and the sections were incubated at room temperature for 30 min. Then, diluted primary antibody (dilution ratio of 1:300) was added dropwise, and the sections were incubated in a wet box at 4 °C overnight (15 h) and then washed 3 times with PBS (3 min per wash). After the sections were dried with absorbent paper, HRP-labeled goat anti-rabbit secondary antibody (dilution ratio of 1:400) was added dropwise, and the sections were incubated for 50 min at room temperature in the dark. TSA fluorescein reagent (prepared in poly(butylene succinate‐co‐butylene terephthalate) (PBST) containing 0.003% H_2_O_2_) was added dropwise to the tissues, and the sections were incubated at room temperature for 20 min. Nuclei were counterstained with DAPI. The sections were mounted with anti-fluorescence quenching mounting medium. Images were observed and captured under a fluorescence microscope, and the mean fluorescence intensity (MFI) was quantitatively analyzed by ImageJ software.

#### Western blot (WB) analysis of AR and EMT-related protein expression in xenograft tumor tissues

Clean tissue samples (size of a rice grain) were lysed on ice in RIPA buffer to extract proteins. The protein concentration was determined by the BCA method. Total protein was separated by sodium dodecyl-sulfate polyacrylamide gel electrophoresis (SDS-PAGE) and transferred to polyvinylidene fluoride (PVDF) membranes. PVDF membranes were soaked in TBST (containing 5% skim milk powder) and incubated at 4 °C overnight. The dilutions of anti-AR and EMT-related protein antibodies and anti-GAPDH (internal control) antibody are shown in Table 1. After adding the HRP-labeled secondary antibody, the membranes were incubated at room temperature for 2.5 h. ECL for routine immunoblotting was used for color development; the bands were scanned, and the gray values of the bands were quantitatively analyzed.

**Table 1:** Dilutions for Primary Antibodies used for Western Blotting

Primary Antibody	Dilution
AR antibody	1:500
p-AR antibody	1:1000
PARP1 antibody	1:1000
CYP17A1 antibody	1:1000
E-cadherin antibody	1:500
N-cadherin antibody	1:500
Vimentin antibody	1:500
α-SMA antibody	1:1000
β-catenin antibody	1:500
ZO-1 antibody	1:400
β-actin antibody (internal reference)	1:1000
GAPDH antibody (internal reference)	1:1000

#### IHC analysis of the expression of MMP2, MMP9, uPA, and SPHK-2 in tumor tissues

Tumor samples were fixed with 4% paraformaldehyde, dehydrated after 72 h, embedded in paraffin, sectioned (5-μm thick), and dehydrated in gradient xylene and ethanol. Tissue sections were placed in a container filled with citric acid-based retrieval solution (0.01 mol/L) and boiled in a microwave for 5 min under high heat, after which the container was placed at room temperature for 10 min; this process was repeated 3 times. After the retrieval solution cooled to room temperature, the container was rinsed 3 times with PBS 3–5 min per wash, and 3% H2O2 was added dropwise to the tissue sections to block endogenous peroxidase. Goat serum working solution was added dropwise, and the sections were blocked at room temperature for 30 min. Then, diluted primary antibody (dilutions are shown in Table 2) was added dropwise, and the sections were incubated in a wet box at 4 °C overnight (15 h), followed by 3 washes with PBS (3 min per wash). After the sections were dried with absorbent paper, HRP-labeled goat anti-rabbit secondary antibody (dilution of 1:400) was added dropwise, and the sections were incubated for 30 min at room temperature. The samples were developed with freshly prepared diaminobenzidine (DAB) solution, nuclei were counterstained with Mayer hematoxylin, and the sections were dehydrated and cleared with gradient ethanol and xylene and mounted with neutral gum. The average optical density (AOD) was quantitatively analyzed by ImageJ software.

**Table 2:** Dilutions for Primary Antibodies used for IHC

Primary Antibody	Dilution
Anti-SPHK2	1:100
Anti-MMP2	1:200
Anti-MMP9	1:200
Anti-uPA	1:100

### Statistical analysis

Data were analyzed using IBM SPSS 26.0, and figures were plotted using GraphPad Prism 8.0. The rank sum test was used to analyze the qualitative data. Quantitative data are expressed as the mean ± standard deviation (x ± s). Comparisons of quantitative data between two samples was performed using the *t* test. For quantitative data with a normal distribution and homogeneity of variances, intergroup comparisons between multiple groups were performed by analysis of variance (ANOVA); the nonparametric rank sum test was performed for data with a non-normal distribution or non-homogeneity of variance. The difference was considered statistically significant when *P* < 0.05.

## Results

### Effect of Lathyrol on the body weight and RCC xenografts of mice

**Fig. 1:**
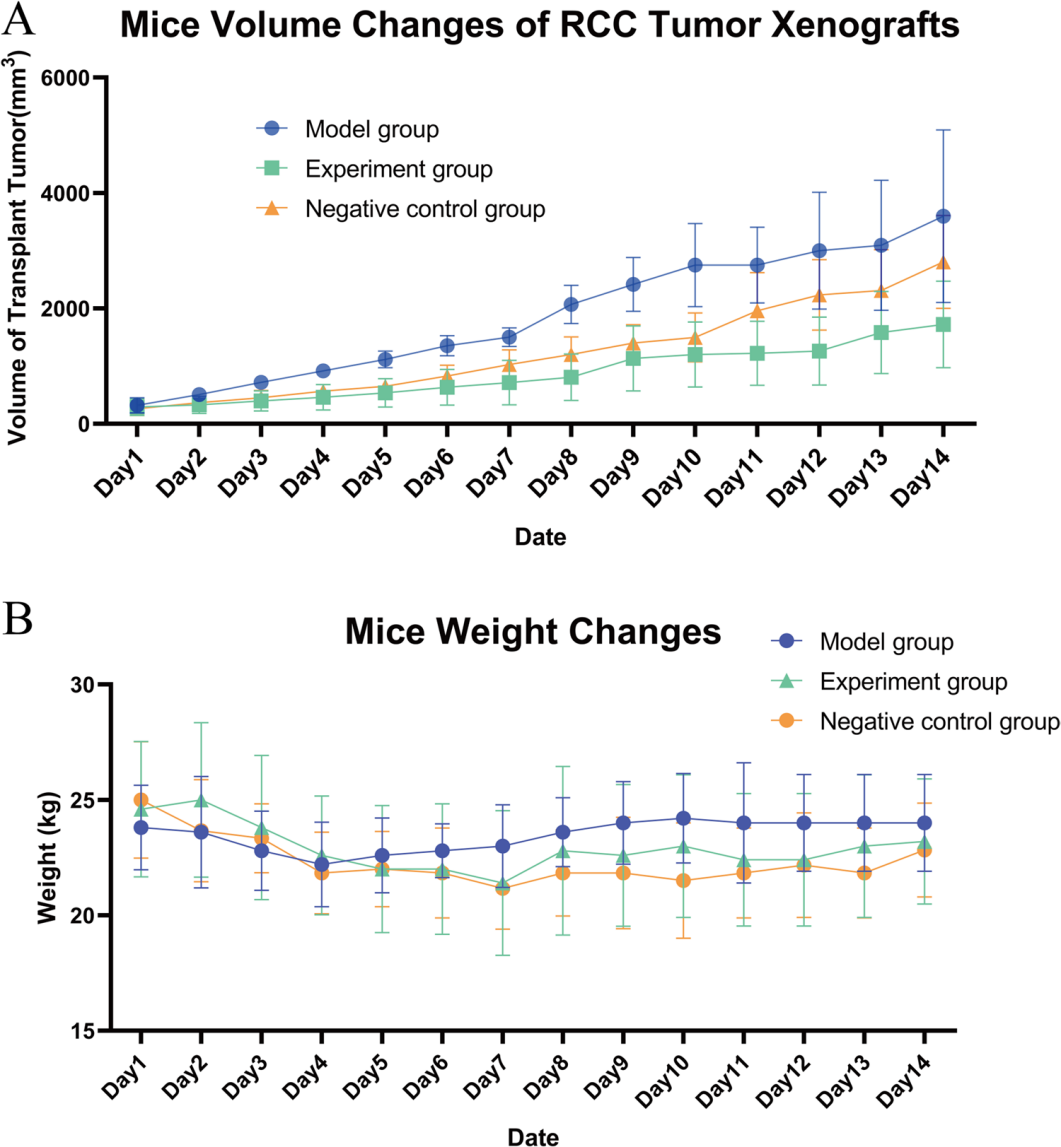
**A** shows the changes in xenograft tumor volume in RCC mice. There were 5 mice in each group. The data satisfied the normality test. The difference between the experiment and model groups was statistically significant (*t* = 3.186, *P* < 0.01); the difference between the model and negative control groups was not statistically significant (*t* = -1.725, *P* > 0.05); and there was no significant difference between the experiment and negative control groups (*t* = 1.503, *P* > 0.05). **B** shows the changes in body weight in RCC mice. The data satisfied the normality test. The difference between the model and negative control groups was statistically significant (*t* = 3.486, *P* < 0.01); the difference between the experiment and negative control groups was not statistically significant (*t* = 1.519, *P* > 0.05); and the difference between the experiment and model groups was not statistically significant (*t* = 0.386, *P* > 0.05)

As seen in Fig. 1A and B, after 14 days of treatment, Lathyrol and cisplatin inhibited the proliferation of RCC xenografts. The difference in tumor volume between the experiment and model groups was statistically significant (*P* < 0.05), but the difference between the experiment and negative control groups was not statistically significant (*P* > 0.1). The effects of Lathyrol and cisplatin on the body weight of the mice were less significant; the difference in body weight between the experiment and model groups was not statistically significant (*P* > 0.1), but the difference between the experiment and negative control groups was statistically significant (*P* < 0.01).

### Lathyrol inhibited the expression and phosphorylation of AR in xenografts in RCC mice

**Fig. 2:**
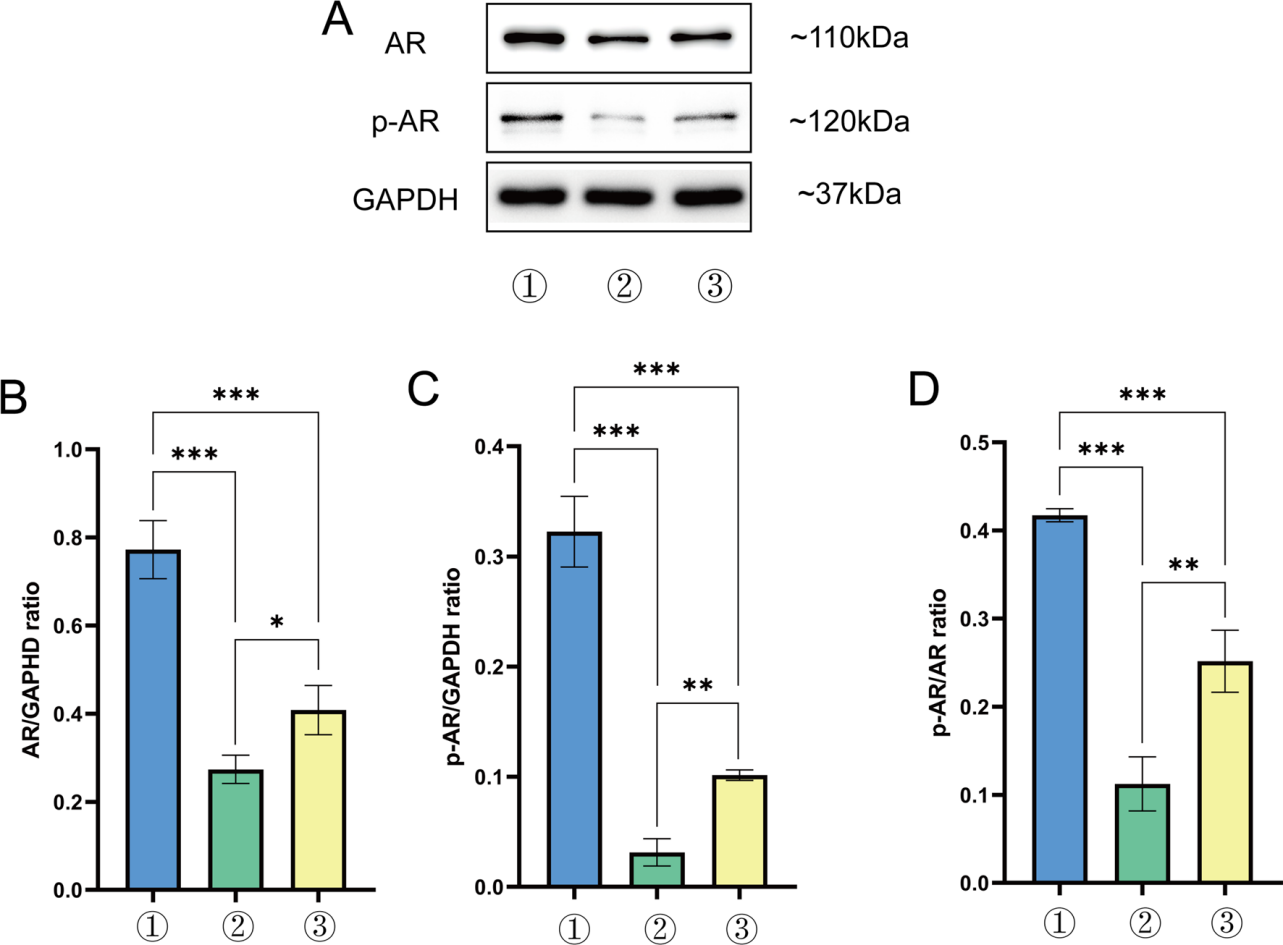
① is the model group, ② is the experiment group, and ③ is the negative control group. **A** shows the expression levels of AR, p-AR, and the internal reference protein GAPDH. The gray value results are shown in Tables 3, 4, and 5. **B** shows the statistical results for the AR/GAPDH gray value ratio; **C** shows the statistical results for the p-AR/GAPDH gray value ratio; and **D** shows the statistical results for the p-AR/AR gray value ratio

The WB results are shown in Fig. 2A and the statistical analyses are shown in Fig. 2B–D and Tables 3, 4, 5. The expression levels of AR and p-AR in RCC xenografts were significantly higher in the model group than in the experiment group (*P* < 0.01) and negative control group (*P* < 0.01). Therefore, Lathyrol and cisplatin inhibited AR expression and phosphorylation in RCC xenografts.

**Table 3:** Analysis of the AR/GAPDH ratio in RCC xenografts

Group	AR/GAPDH ratio
Model group	0.7724 ± 0.0381
Experiment group	0.2734 ± 0.0184
Negative control group	0.4082 ± 0.0323

This table is a summary of Fig. 2A, B

The gray values for the WB results were analyzed by Image-Pro-Plus (IPP) software

**Table 4:** Analysis of the p-AR/GAPDH ratio in RCC xenografts

Group	p-AR/GAPDH ratio
Model group	0.3225 ± 0.0185
Experiment group	0.0313 ± 0.0071
Negative control group	0.1014 ± 0.0028

This table is a summary of Fig. 2A, C

The gray values for the WB results were analyzed by Image-Pro-Plus (IPP) software

**Table 5:** Analysis of the p-AR/AR ratio in RCC xenografts

Group	p-AR/AR ratio
Model group	0.4172 ± 0.0043
Experiment group	0.1124 ± 0.0177
Negative control group	0.2516 ± 0.0203

This table is a summary of Fig. 2A, D

The gray values of the WB results were analyzed by Image-Pro-Plus (IPP) software

### Lathyrol inhibited the expression levels of AR and the key enzymes CYP17A1 and PARP1 in RCC xenografts

**Fig. 3:**
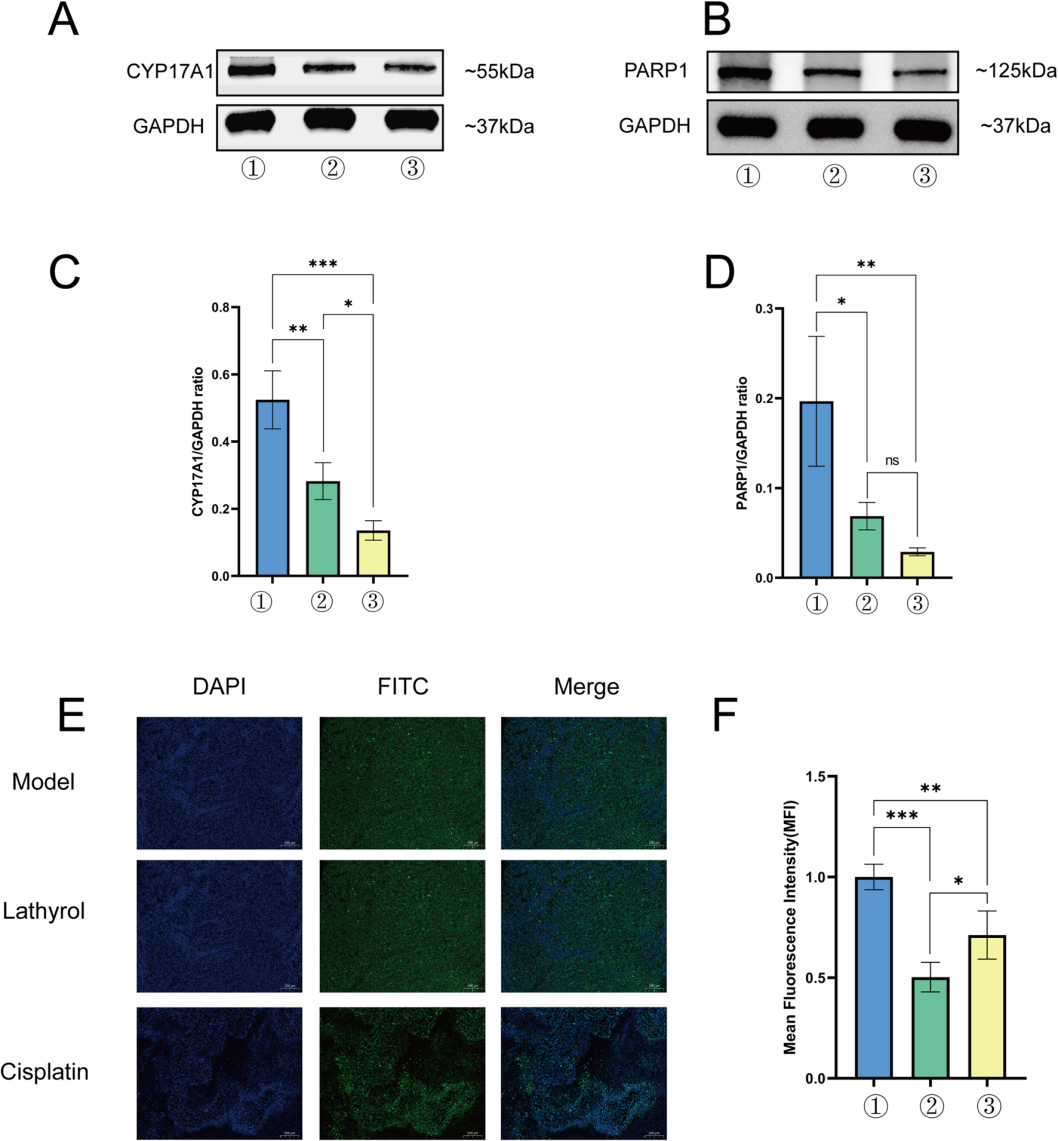
① is the model group, ② is the experiment group, and ③ is the negative control group. **A** shows the expression levels of CYP17A1 and the internal reference protein GAPDH, and the gray value results are shown in Table 6. **B** shows the expression levels of PARP1 and the internal reference protein GAPDH. The gray value results are shown in Table 7. **C** shows the statistical results for the CYP17A1/GAPDH gray value ratio. **D** shows the statistical results for the PARP1/GAPDH gray value ratio. **E** shows the IF-AR staining results for mouse xenografts. DAPI nuclear staining is blue, and FITC staining for the AR marker is green. The MFI results are shown in Table 8. **F** shows the statistical results for the MFI

The IF results are shown in Fig. 3E. AR were mainly expressed in the cytoplasm, with a small amount of AR expression in the nucleus. The WB results are shown in Fig. 3A, B and the statistical analyses are shown in Fig. 3C, D, F and Tables 6, 7, 8. The expression levels of CYP17A1 and PARP1 in RCC xenografts was significantly higher in the model group than in the experiment group (*P* < 0.01) and negative control group (*P* < 0.01). Therefore, Lathyrol and cisplatin inhibited the expression levels of AR and the key enzyme CYP17A1 in RCC xenografts.

**Table 6:** Analysis of the CYP17A1/GAPDH ratio in RCC xenografts

Group	CYP17A1/GAPDH ratio
Model group	0.5243 ± 0.0497
Experiment group	0.2823 ± 0.0317
Negative control group	0.1356 ± 0.0168

This table is a summary of Fig. 3A, C

The gray values for the WB results were analyzed by Image-Pro-Plus (IPP) software

**Table 7:** Analysis of the PARP1/GAPDH ratio in RCC xenografts

Group	PARP1/GAPDH ratio
Model group	0.1966 ± 0.0418
Experiment group	0.0688 ± 0.0088
Negative control group	0.0288 ± 0.0025

This table is a summary of Fig. 3B, D

The gray values for the WB results were analyzed by Image-Pro-Plus (IPP) software

**Table 8:** MFI analysis of AR expression in RCC xenografts

Group	Mean fluorescence intensity of AR
Model group	1.00 ± 0.0365
Experiment group	0.5024 ± 0.0423
Negative control group	0.6504 ± 0.0144

This table is a summary of Fig. 3E, F

The MFI of AR was analyzed by ImageJ software

### Lathyrol inhibited SPHK-2 expression in RCC xenografts in mice

**Fig. 4:**
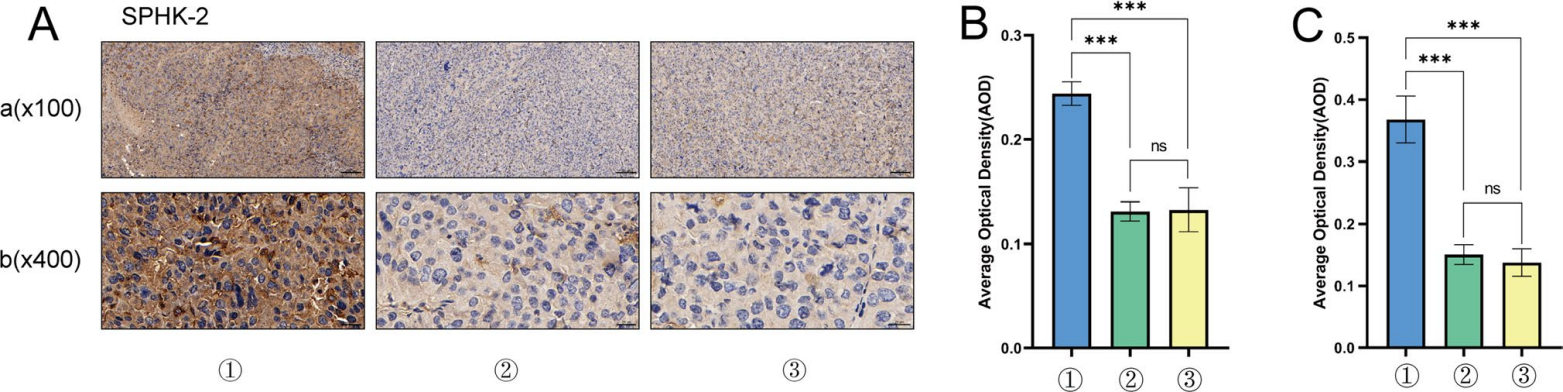
① is the model group, ② is the experiment group, and ③ is the negative control group. **A** shows the IHC staining results for SPHK-2 expression, visualized with DAB; positive expression is indicated by brown granules. a shows the observation results under low magnification (× 100), and b shows the results under high magnification (× 400). Table 9 shows the AOD analysis results. **B** shows the AOD statistical analysis results for SPHK-2 expression under low magnification. **C** shows the AOD statistical analysis results for SPHK-2 expression under high magnification

The IHC results are shown in Fig. 4A and the statistical analyses are shown in Fig. 4B, C and Table 9. The expression of SPHK-2 was mainly observed in the cytoplasm, with a small amount of SPHK-2 expression in the nucleus. The expression of SPHK-2 was significantly higher in the model group than in the experiment group (*P* < 0.001) and negative control group (*P* < 0.001), and the difference between the experiment and negative control groups was not statistically significant (*P* > 0.05). Therefore, Lathyrol and cisplatin significantly inhibited SPHK-2 expression in RCC xenografts.

**Table 9:** AOD analysis of SPHK-2 expression in RCC xenografts

	Low magnification (× 100)	High magnification (× 400)
Model group	0.2439 ± 0.0065	0.3680 ± 0.0219
Experiment group	0.1311 ± 0.0053	0.1503 ± 0.0092
Negative control group	0.1325 ± 0.0120	0.1376 ± 0.0128

This table is a summary of Fig. 4A–C

The AOD of SPHK-2 was analyzed by ImageJ software

### Lathyrol promoted the expression of E-cadherin and ZO-1 and inhibited the expression of N-cadherin, β-catenin, vimentin and α-SMA in RCC mice

**Fig. 5:**
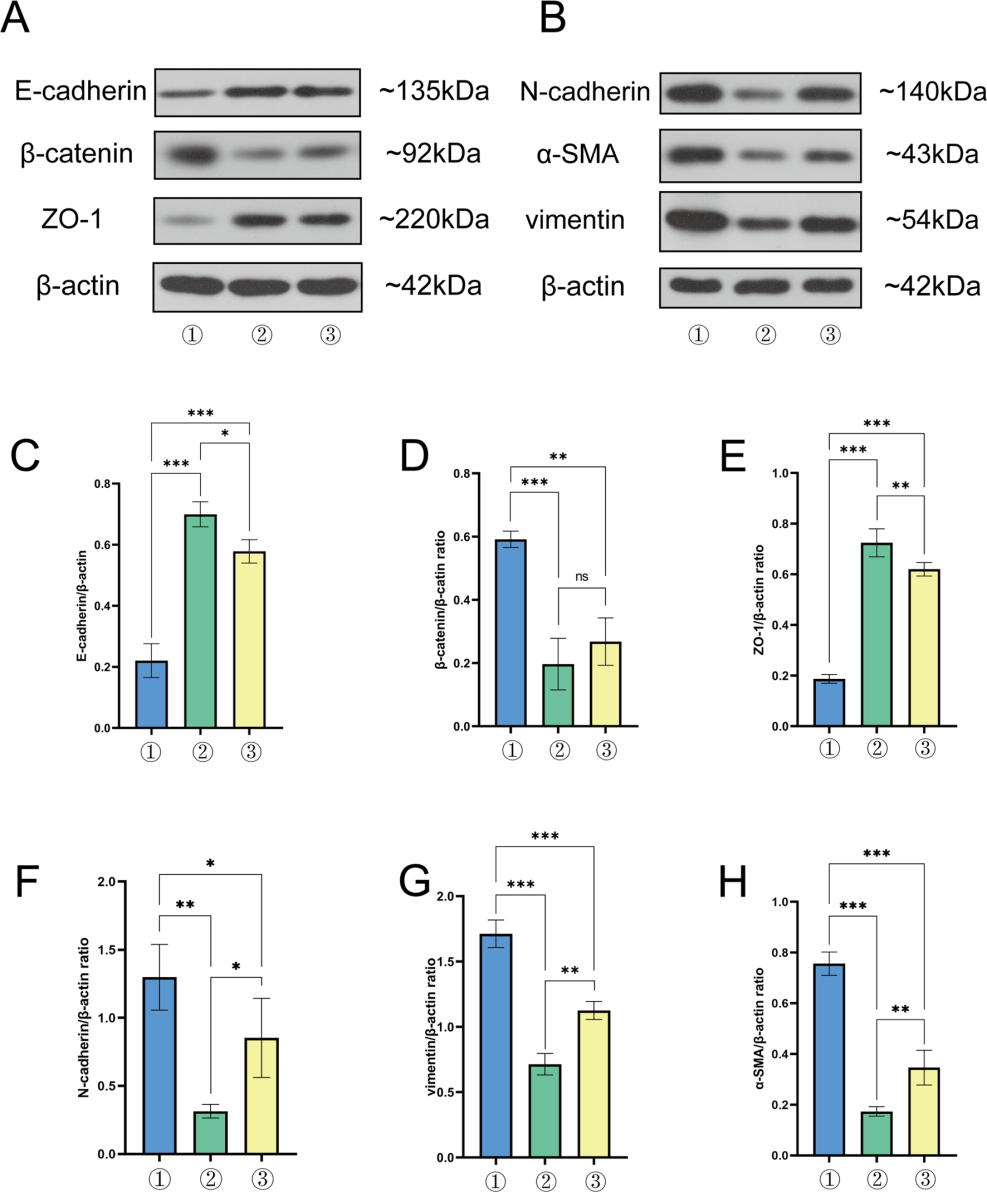
① is the model group, ② is the experiment group, and ③ is the negative control group. **A** shows the expression levels of epithelial biomarkers and the internal reference protein β-actin, which were detected by WB, and gray value analysis was performed using ImageJ software, with results shown in Table 10. **B** shows the expression levels of mesenchymal biomarkers and the internal reference protein β-actin, and gray value analysis was performed using ImageJ software, with results shown in Table 11. **C** shows the statistical analysis results for the gray value of E-cadherin/β-actin. **D** shows the statistical analysis results for the gray value of β-catenin/β-actin. **E** shows the statistical analysis results for the gray value of ZO-1/β-actin. **F** shows the statistical analysis results for the gray value of N-cadherin/β-actin. **G** shows the statistical analysis results for the gray value of vimentin/β-actin. **H** shows the statistical analysis results for the gray value of α-SMA/β-actin

The results are shown in Fig. 5A, B and the statistical analyses are shown in Fig. 5C–H and Tables 10, 11. In the model group, the expression levels of the epithelial biomarkers E-cadherin and ZO-1 were decreased, the expression of β-catenin was increased, and the expression of the mesenchymal biomarker N-cadherin was increased in the RCC xenografts. Compared with those in the model group, the expression levels of the epithelial biomarkers E-cadherin and ZO-1 were higher, the expression of β-catenin was lower, and the expression levels of the mesenchymal biomarkers N-cadherin, vimentin and α-SMA were higher in the experiment and negative control groups.

**Table 10:** Gray value analysis of epithelial biomarker expression in RCC xenografts

Group	E-cadherin/β-actin	β-catenin/β-actin	ZO-1/β-actin
Model group	0.2204 ± 0.0320	0.5914 ± 0.0150	0.1867 ± 0.0099
Experiment group	0.6995 ± 0.0237	0.1965 ± 0.0471	0.7243 ± 0.0319
Negative control group	0.5781 ± 0.0221	0.2677 ± 0.0433	0.6201 ± 0.0154

This table is a summary of Fig. 5A, C, D, E

The gray values for the WB results were analyzed by ImageJ software

**Table 11:** Gray value analysis of mesenchymal biomarker expression in RCC xenografts

Group	N-cadherin/β-actin	vimentin/β-actin	α-SMA/β-actin
Model group	1.2975 ± 0.1396	1.7118 ± 0.0606	0.7561 ± 0.0266
Experiment group	0.1344 ± 0.0288	0.7135 ± 0.0473	0.1736 ± 0.0108
Negative control group	0.8524 ± 0.1677	1.1250 ± 0.0396	0.3462 ± 0.0394

This table is a summary of Fig. 5B, F, G, H

The gray values for the WB results were analyzed by ImageJ software

### Lathyrol inhibited the invasiveness of xenografts in RCC mice

**Fig. 6:**
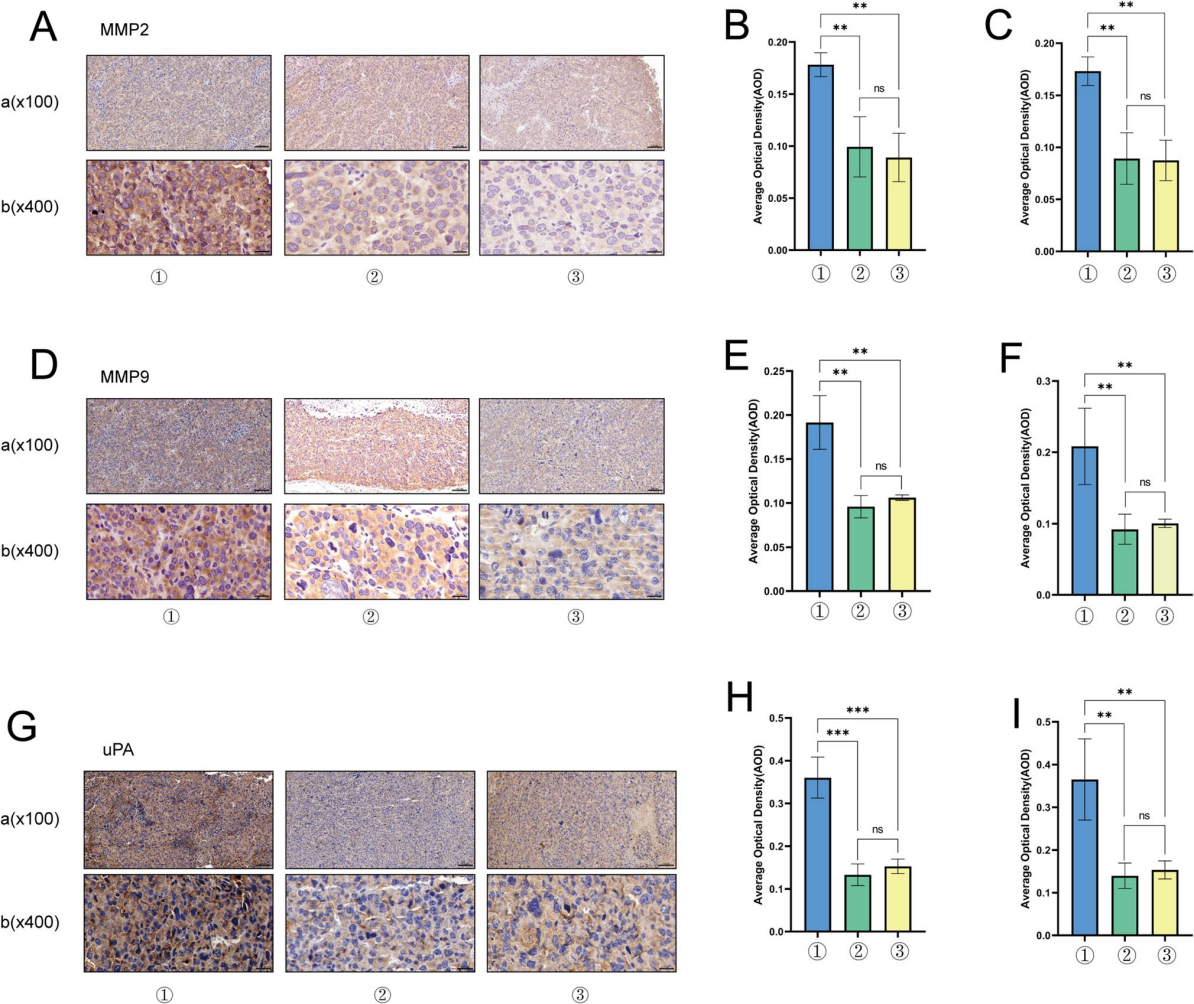
① is the model group, ② is the experiment group, and ③ is the negative control group. **A** shows the IHC results for MMP2, visualized by DAB, with positivity indicated by brown granules. a shows the result under low magnification (× 100), and b shows the results under high magnification (× 400). AOD was analyzed by ImageJ software, and the results are shown in Table 12. **B** shows the statistical analysis results for the AOD of MMP2 expression under low magnification. **C** shows the statistical analysis results for the AOD of MMP2 expression under high magnification. **D** shows the IHC results for MMP9, visualized by DAB staining, and positivity is indicated by brown granules. a shows the result under low magnification (× 100), and b shows results under high magnification (× 400). AOD was analyzed using ImageJ software, and the results are shown in Table 13. **E** shows the statistical analysis results for the AOD of MMP9 expression under low magnification. **F** shows the statistical analysis results for the AOD of MMP9 expression under high magnification. **G** shows the IHC staining results for uPA, visualized with DAB, with positivity indicated by brown granules. a shows the result under low magnification (× 100), and b shows results under high magnification (× 400). AOD analysis was performed using ImageJ software, and the results are shown in Table 14. **H** shows the AOD statistical analysis results for uPA expression under low magnification. **I** shows the AOD statistical analysis results for uPA expression under high magnification

The IHC results are shown in Fig. 6A, D, G and the statistical analyses are shown in Fig. 6B, C, E, F, H, I and Tables 12, 13, 14). The expression levels of MMP2, MMP9, and uPA in RCC xenografts were significantly higher in the model group than in the experiment and negative control groups; the differences in the expression levels of MMP2, MMP9, and uPA between the experiment and negative control groups were not statistically significant.

**Table 12:** AOD analysis of MMP2 expression in RCC xenografts

	Low magnification (× 100)	High magnification (× 400)
Model group	0.1834 ± 0.0033	0.1771 ± 0.0078
Experiment group	0.0993 ± 0.0167	0.0892 ± 0.0143
Negative control group	0.0891 ± 0.0134	0.0874 ± 0.0112

This table is a summary of Fig. 6A–C

The AOD of MMP2 was analyzed by ImageJ software

**Table 13:** AOD analysis of MMP9 expression in RCC xenografts

	Low magnification (× 100)	High magnification (× 400)
Model group	0.1916 ± 0.0177	0.1856 ± 0.0202
Experiment group	0.0959 ± 0.0072	0.0921 ± 0.0121
Negative control group	0.1061 ± 0.0018	0.1005 ± 0.0034

This table is a summary of Fig. 6(D, E&F). The AOD of SPHK-2 was analyzed by ImageJ software

**Table 14:** AOD analysis of uPA expression in RCC xenografts

	Low magnification (× 100)	High magnification (× 400)
Model group	0.3605 ± 0.0277	0.3653 ± 0.0549
Experiment group	0.1330 ± 0.0146	0.1396 ± 0.0173
Negative control group	0.1527 ± 0.0098	0.1533 ± 0.0122

This table is a summary of Fig. 6G–I

The AOD of SPHK-2 was analyzed by ImageJ software

## Discussion

The early clinical symptoms of RCC patients are atypical, but the late-stage symptoms may be painless hematuria, low back pain, and abdominal mass. Because the location of RCC in kidney is not obvious and early symptoms are not apparent, 30–40% of RCC patients have already developed metastasis at the first diagnosis. However, nearly 30% of RCC patients who undergo surgical treatment have recurrence and metastatic renal cell carcinoma (mRCC) [12]. Currently, radical nephrectomy is still the main clinical treatment for RCC [13]. The prognosis of early-stage RCC patients who undergo surgical treatment is good, but for advanced renal cell carcinoma (aRCC) patients who are insensitive to radiotherapy and chemotherapy [7], surgical treatment can only reduce the tumor burden or improve symptoms; therefore, surgical treatment can only be used as a palliative treatment [14]. As such, it is important to explore effective treatments for RCC. TCM, in terms of antitumor efficacy, has high economic benefits, significant clinical efficacy, and fewer side effects and toxicity [15]. Lathyrol is the dry mature seed of *Euphorbia lathyris* L. and is a TCM with a variety of active antitumor and anti-drug resistance components. Lathyrol can not only directly kill cancer cells and inhibit the proliferation of tumor tissues but also regulate the tumor microenvironment (TME) to inhibit the drug resistance of tumor cells, thereby increasing the efficacy of anticancer drugs [16]. As the results showed, Lathyrol have little effects on the body weights of mice and can inhibit the proliferation of RCC xenografts (as shown in Fig. 1).

**Fig. 7:**
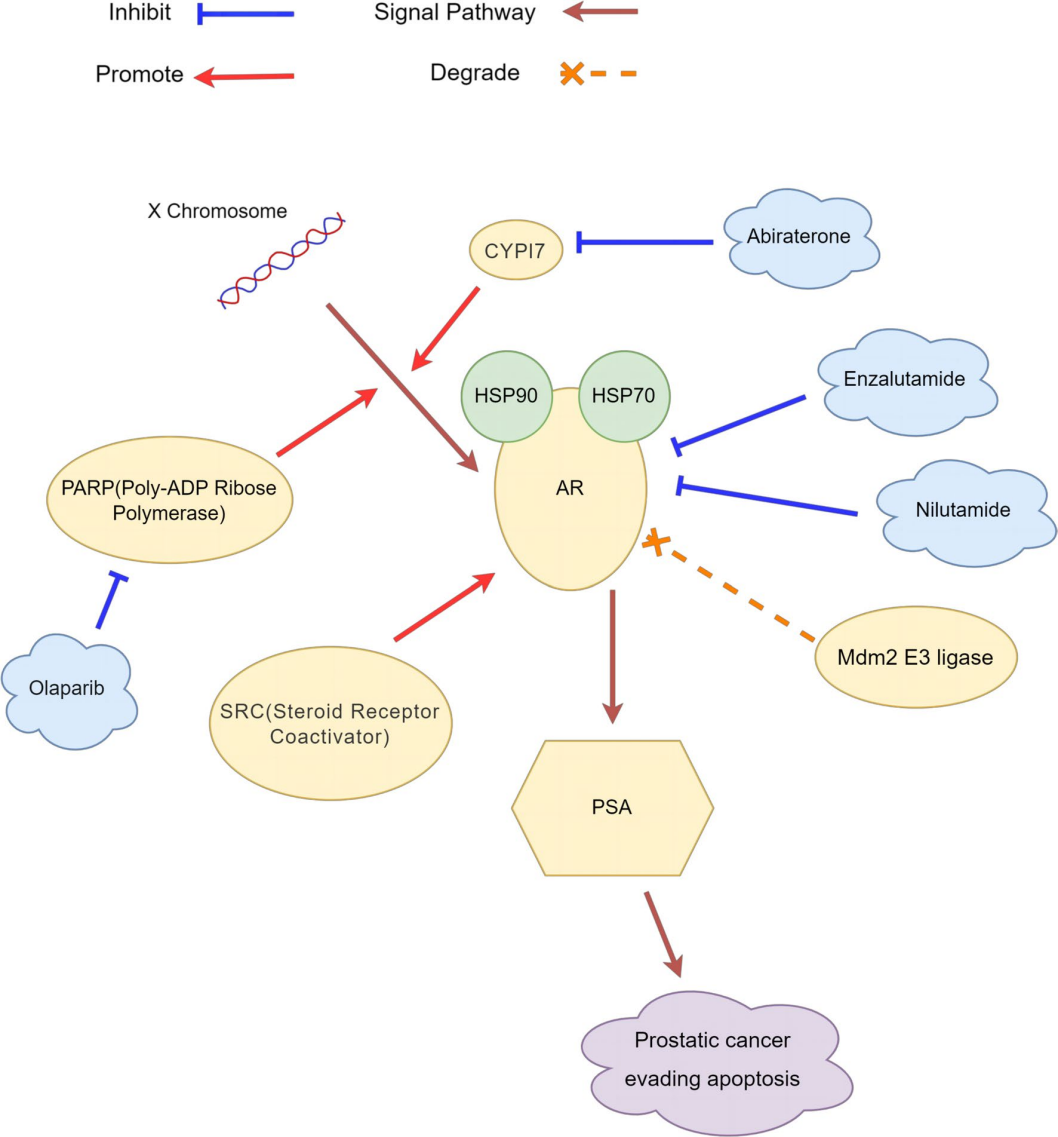
Schematic diagram of AR-related targets and corresponding target drugs

From the results, we found that Lathyrol may generate the effect to the expression of CYP17A1 and PARP1 (as shown in Fig. 3A, B, C, D and Tables 6, 7), thereby affecting the synthesis and phosphorylation of AR (as shown in Fig. 2, 3E, F and Tables 3–5, 8), which has never been showed before. Studies have shown that AR activation and expression promote the occurrence and development of tumors in the advanced stage and promote malignant behaviors, such as invasion, spread and proliferation, and tumor drug resistance through different mechanisms, such as inhibiting the secretion of relevant immune factors to promote tumor immune escape, inducing angiogenesis, and activating EMT [17]. CYP17A1 is one of the key enzymes that affect AR synthesis, and its inhibitor, abiraterone, has been one of the targeted drugs recently implemented by international medical institutions for the treatment of metastatic castration-resistant prostate cancer (mCRPC) [18]. Liu et al. [19] found that CYP17A1 is expressed in rat testis and other reproductive organs and that the upregulation of CYP17A1 expression promotes AR expression in testis tissue. Giatromanolaki et al. [20] found that CYP17A1 was overexpressed in the cytoplasm of PCa cells and was directly related to AR expression in cancer cell nuclei and that the application of abiraterone, in addition to inhibiting CYP17A1 expression, could also block the secretion of AR and thus block the nuclear accumulation of AR. Poly ADP-ribose polymerase-1 (PARP-1) plays an important role in the DNA damage repair process and functions as a specific regulator of the upstream and downstream targets of transcription factors. However, PARP-1 also plays a role in promoting tumor progression in several tumor models [21]. Clinical anticancer drugs developed against PARP, i.e., PARP inhibitors such as olaparib, are also the targeted drugs currently used for the diagnosis and treatment of PCa patients [22]. Xie et al. [23] found that metformin induced the cleavage and degradation of PARP1, inhibited the expression levels of AR and its splice variant AR-V7, and thus induced the death of PCa cells. Therefore, as one of the important targets of AR synthesis and a participant in AR signal transduction, PARP1 has potential in tumor treatment and may provide an effective treatment strategy for mCRPC and mRCC patients. At present, through research on each key target associated with AR (as shown in Fig. 7), the corresponding drugs for each site of action have been developed and applied in the treatment of patients with PCa at each stage [24]. Although the research on AR in PCa is relatively mature, studies on AR in RCC are rare. In recent years, AR, as a classic immune target, has been studied with regard to the treatment of RCC. Bialek et al. [25] found that compared with normal physiological tissues, in tumor tissues, AR and its splice variant AR-V7RCC were more frequently expressed. Lee et al. [26] found that the inhibition of lysine-specific histone demethylase 1 (LSD1) expression inhibited the binding of AR to downstream target genes, thereby inhibiting the growth state and migration ability of RCC. Therefore, AR is an important target that affects the activity and function of RCC cells. The results of this study showed that while the expression of the key enzymes CYP17A1 and PARP1 was inhibited, the synthesis and phosphorylation of AR were also inhibited. Therefore, these two enzymes may be related to the synthesis and phosphorylation of AR to some extent. It is possible that Lathyrol may influence the expression of CYP17A1 and PARP1, thereby affecting the synthesis and phosphorylation of AR, then produce negative results to the function and activity of neoplasm cells.

The function of sphingosine kinase is to phosphorylate sphingosine to sphingosine 1-phosphate (S1P) to maintain the balance of sphingolipid metabolites [27]. There are two isoforms of sphingosine kinase: sphingosine kinase 2 (SPHK1) and SPHK2. Although SPHK1 and SPHK2 share high sequence similarity, the distribution, regulation and functions of SPHK2 are different from those of SPHK2 [28], and SPHK-2 can be located in the nucleus and organelles of physiological cells. In the nucleus, SPHK-2 regulates gene expression and maintains telomere integrity; in organelles, such as the endoplasmic reticulum and mitochondria, SPHK-2 regulates the apoptosis pathway [6, 27]. The overexpression of SPHK2 has a tumor-promoting effect; that is, it can promote the proliferation, migration and invasion of tumor cells. Lan et al. [29] found that the overexpression of SphK2 promoted the proliferation of adipocyte-induced epithelial ovarian cancer (EOC) cells. Huo et al. [30] found that methylated SPHK2 promoted the proliferation, migration and invasion of gastric cancer (GC) cells through the inhibition of KLF2 expression. Shi et al. [31] found that advanced hepatocellular carcinoma (HCC) overexpressed SPHK-2 and developed resistance to the anticancer drug regorafenib and that silencing SPHK-2 expression restored the sensitivity of HCC to regorafenib. The results of this study showed that Lathyrol may affect the invasion and EMT activity of RCC xenografts by affecting the secretion of SPHK-2(as shown in Fig. 4 and Table 9).MMPs and uPA promote tumor infiltration and invasion in the TME. MMP degrades extracellular matrix (ECM) components and releases matrix factors, cell surface-bound cytokines, and growth factors or their receptors to affect tissue integrity, immune cell recruitment and tissue turnover and promote tumor cell migration and invasion [32]. Masucci et al. [33] found that increased levels of uPA and uPAR in tumor tissues, stroma and biological fluids were correlated with unfavorable clinicopathological features and poor patient prognosis. After uPA binds to uPAR, it activates plasminogen to plasmin. Plasmin is a broad-spectrum matrix and fibrin-degrading enzyme that promotes tumor cell invasion and spread to distant sites. Chien et al. [34] found that timosaponin AIII suppressed the migration and invasion ability of cervical cancer cells by inhibiting the expression of uPA. This study determined the invasiveness of RCC xenografts by assessing the expression of xenograft-associated invasion proteins. The results showed that Lathyrol may affect the invasiveness of RCC xenografts(as shown in Fig. 6 and Table 12–14) by affecting the expression of AR and SPHK2.

The trans-differentiation process of epithelial cells into mesenchymal cells is called EMT, which is an important part of normal physiological functions, such as embryonic development and wound healing [35]. In the process of EMT, there are two types of molecular markers: epithelial biomarkers and mesenchymal biomarkers. The ratio changes in these two indicators reflect the EMT process of cells and tissues. Common epithelial biomarkers include E-cadherin, β-catenin, and ZO-1, and common mesenchymal biomarkers include N-cadherin, vimentin, and α-SMA [36]. However, in the TME, EMT plays an important role in tumor progression, metastasis and drug resistance. EMT of tumor cells could lead to the loss of cell polarity, promote tumor migration, invasion and infiltration, cause tumor cells to develop immune evasion, and promote progression into advanced and metastatic diseases [37]. Therefore, the analysis of EMT in tumor cells and tumor tissues is an important means to determine cancer progression. In this study, the EMT ability of RCC xenografts was analyzed by assessing the expression of xenograft-associated EMT marker proteins. The results showed that Lathyrol can affect the EMT of RCC xenografts (as shown in Fig. 5 and Tables 10, 11), an effect that may be associated with the inhibition of the expression of AR and SPHK2.

We have been committed to conducting research on the mechanism of action of the TCM Lathyrol in human RCC 786-O cells and Renca cells. In recent years, Lathyrol has been found to act through various pathways, such as the inhibition of the NF-κB pathway and related proteins in cancer tissues, promote the apoptosis of RCC cells and inhibit the proliferation and invasion of Renca cells to a certain extent by inhibiting the expression and activation of proteins in the TGF-β/Smad pathway. In summary, based on previous studies by our group, in this study, Lathyrol inhibited the expression of invasive proteins and the incidence of EMT in RCC xenografts through the inhibition of the expression of AR and SPHK-2, key proteins in RCC xenografts, thus significantly inhibiting the invasion and EMT ability of RCC xenografts in mice; notably, Lathyrol had a small effect on the body weight of the mice. However, due to the lack of a specific gene level analysis and the analyses of other possible targets of Lathyrol, other pathways and related molecular mechanisms were not explored in depth. Therefore, the mechanism of action and specific targets associated with the development of RCC still need to be explored further to provide new ideas for the clinical diagnosis and treatment of patients with RCC.

## Conclusion

Lathyrol and cisplatin inhibit the proliferation of RCC xenografts, reduce the protein expression levels of AR, CYP17A1, SPHK2, PARP1, E-cadherin, and ZO-1 in tumor tissues (*P* < 0.05), and promote the protein expression levels of N-cadherin, β-catenin, vimentin and α-SMA (*P* < 0.05). Therefore, Lathyrol reduces RCC invasion and EMT by affecting the expression of AR and SPHK2 in RCC mice.

### Supplementary Information

Additional file 1.

## Data Availability

The authors declare that the data supporting the findings of this study are available within the paper and its Supplementary Information files. Should any raw data files be needed in another format they are available from the corresponding author upon reasonable request.
