# Supplementary material for: Lathyrol reduces the RCC invasion and incidence of EMT via affecting the expression of AR and SPHK2 in RCC mice
Source: Discov Oncol. 2024 Jul 4;15:264. doi: 10.1007/s12672-024-01130-w (PMC11224167; doi:10.1007/s12672-024-01130-w)
Supplement: Supplementary file 1 — Additional file 1. [file 12672_2024_1130_MOESM1_ESM.pdf]

Lathyrol Reduces the RCC Invasion and Incidence of EMT via Affecting the Expression of AR and SPHK2 in RCC mice

Shengyou Song[1], Lunwei Tai[1], Yuqi Xu[1], Junling Jiang[2], Lei Zhou[2], Junfeng Zhao[2]#

1.Department of Urology, The Second Affiliated Hospital of Henan University of Chinese Medicine, Zhengzhou, Henan 450002, China.

2.Department of Urology, The Second Affiliated Hospital of Henan University of Chinese Medicine, Dongfeng Road 6#, Zhengzhou, Henan 450002, China.

#Correspondence to Dr. Junfeng Zhao, Department of Urology, The Second Affiliated Hospital of Henan University of Chinese Medicine, Dongfeng Road 6#, Zhengzhou, Henan 450002, China. Tel: 86-371-60908781; Fax:86-371-60908800. Email: zhaojunfeng@hactcm.edu.cn

Correspondence author:
Junfeng Zhao, M.D., Ph.D.
Department of Urology, The Second Affiliated Hospital of Henan University of Chinese Medicine, Zhengzhou, Henan, People's Republic of China, 450002
Tel: +86-13526870287
Fax: +86-371-60908800
E-mail: zhaojunfeng@hactcm.edu.cn

Checklist

1. Article documents:

* All required documents such as papers, abstracts, graphs and tables have been uploaded.

* All document formats and file types have been checked for compliance with journal requirements.

* The file names and order of all documents have been verified.

2. Author Information:

* The names, affiliations, titles and contact details of all authors have been provided.

* The contributions and extent of contributions of all authors have been recognized.

3. Content of the paper:

* The content of the paper has been checked for conformity with the purpose and scope of the journal.

* It has been confirmed whether the research method, experimental design, data analysis and conclusions of the paper are reasonable.

* The language and presentation of the paper have been checked for accuracy, clarity and coherence.

4. Graphs and tables:

* It has been confirmed that all graphics and tables have been uploaded and meet the requirements of the journal.

* The file type and resolution of the graphics and tables have been checked to meet the journal requirements.

* The file names and order of the graphs and tables have been verified.

5. Citations and references:

* All citations and references in the paper have been checked to meet the format requirements of the journal.

* The order and accuracy of all citations and references have been confirmed.

6. Additional information:

* It has been confirmed that all information has been provided.

CRediT author statement

Shengyou Song: Conceptualization, Methodology, Validation, Formal analysis, Investigation, Resources, Data Curation, Writing - Original Draft, Visualization, Project administration.
Lunwei Tai: Data curation, Investigation.
Yuqi Xu: Supervision.
Junling Jiang: Investigation, Validation.
Lei Zhou: Visualization, Investigation.
Junfeng Zhao#: Conceptualization, Writing- Reviewing and Editing, Supervision, Funding acquisition

Data availability

The authors declare that the data supporting the findings of this study are available within the paper and its Supplementary Information files. Should any raw data files be needed in another format they are available from the corresponding author upon reasonable request. Source data are provided with this paper.

Conflict of interest statement

All authors declare that there are no competing interests.

Animal Ethics Statement

The authors declare that all experimental protocols about this study were approved by the Central Laboratory Ethics Committee of the Second Affiliated Hospital of Henan University of Traditional Chinese Medicine (acceptance number: SL-HNSZYY-2023-002; approval number: PZ-HNSZYY-2023-002). All methods were carried out in accordance with relevant guidelines and regulations. The study conforms to the ARIVE guidelines and should be carried out in accordance with the 1964 Declaration of Helsinki and its later amendments or comparable ethical standards, the U.K. Animals (Scientific Procedures) Act, 1986 and associated guidelines, EU Directive 2010/63/EU for animal experiments, or the National Institutes of Health guide for the care and use of Laboratory animals (NIH Publications No. 8023, revised 1978).

河南省中医院(河南中医药大学第二附属医院)

实验动物福利伦理审查表

申请日期：2023.01.10　　受理编号：SL-HNSZYY-2023-002　批准文号：PZ-HNSZYY—2023-002

课题名称及编号	千金子二萜醇通过TGF-β/Smad信号通路对肾癌小鼠免疫因子 IL-7、IL-10 及 VEGF 表达的影响 20-21YZ2216	课题来源	河南省中医药研究专项课题
课题负责人	赵俊峰	所在科室	泌尿外科
动物实验负责人	宋圣佑	电话	18836936875
		邮箱	ssysonic@126.com
参与实施动物实验的人数	2	经专业培训的人数	2

参与动物实验人员姓名、相关经验、　培训、资质和能力的描述：

宋圣佑接受河南中医药大学实验安全管理与教育平台动物实验上岗培训，并考试合格。

郜伟伦接受河南中医药大学实验安全管理与教育平台动物实验上岗培训，并考试合格。

动物实验设施许可证编号： SYXK（豫）2021-0018

现有动物实验设施条件是否与拟开展动物实验的规范性要求相匹配的描述：

河南省中医院实验动物中心，拥有先进仪器设备，高标准实验平台，近年来承担多项国家自然科学基金项目及各类科研项目，能够满足科研需求。

拟实验时间： 2023 年 2 月 3 日　至　2023 年 5 月 16 日

动物实验项目的目的、意义及如何设计已达成研究目标的：

动物实验目的：根据对比不同组小鼠各项指标的差异，探究肾癌发病的作用机制以及传统中药千金子的抗肿瘤疾病作用及作用机制。

意义：肾癌的发病机制目前尚未完全探明，往期研究表示千金子对于具有较明确的抗肿瘤效应，然而千金子抗肿瘤疾病的相关机制尚未得到充分研究与有力的数据支持。因此，本实验意图通过动物实验来探索肾癌的发生发展机制与千金子发挥抗肿瘤作用所涉及到的靶点来阐述其作用机制。

如何设计：本实验设置 3 个实验组，通过小鼠皮下注射 Renca 细胞造肾癌模型，设置模型组、中药组以及顺铂对照组。

<table>
<tr><td rowspan="5">拟使用动物信息</td><td>动物来源</td><td>斯贝福（北京）生物技术有限公司</td><td>许可证编号</td><td>SCXK（京）-0010</td><td>质量合格证</td><td colspan="2">有☑　无☐</td></tr>
<tr><td>品种/品系</td><td colspan="3">BALB/c 小鼠</td><td>等级</td><td colspan="2">SPF 级</td></tr>
<tr><td>雌(♀)</td><td rowspan="2">数量(只)</td><td></td><td rowspan="2">体重(g)</td><td></td><td rowspan="2">月龄(M)</td><td></td></tr>
<tr><td>雄(♂)</td><td>40</td><td>20</td><td>2</td></tr>
<tr><td colspan="7">选择实验动物种类和数量的原因：
BALB/c 小鼠性情温顺，易于捉取，具有群居优势，便于同笼多个饲养。生长发育迅速，生长速度较快。实验设计设置 3 组实验组，每组 10 只。考虑笼盒大小，预计每笼饲养 5 只小鼠，为保证各组饲养条件一致，以及考虑到饲养过程的损耗，故购入 40 只 BALB/c 小鼠。</td></tr>
<tr><td rowspan="5">拟开展动物实验的详细信息</td><td colspan="7">列出对动物可能造成的伤害，包括动物运输、动物饲养方式、实验操作步骤中等可能产生的伤害或不适以及拟采取的防控措施：
1.运输过程中可能被挤压伤害或者密闭缺氧的可能；2.饲养过程中争抢食物而受伤的可能；3.操作不当或灌胃失误的可能。措施：1.运输过程中要求保持轻拿轻放，运输环境透风、清洁；2.饲养过程按时投放饲料食物，密切观察进食情况，防止争抢；3.手术操作人员进行严格培训考核。</td></tr>
<tr><td colspan="7">主要观察指标：
各实验组肿瘤学指标、瘤块和器官标本中相关差异蛋白，外周血浆中 IL-7、IL-10 和 VEGF 等炎症因子含量。</td></tr>
<tr><td colspan="7">仁慈终点或实验终结的指标：实验中小鼠出现严重的应激反应</td></tr>
<tr><td colspan="3">动物处死方式：
麻醉后采血</td><td colspan="4">非处死动物处置方式：
无</td></tr>
<tr><td colspan="7">是否使用有毒（害）物质（感染、放射、化学毒、其他）☑否　☐是
说明：</td></tr>
<tr><td colspan="8">利害分析的小结，说明为何预期的利益多于害处？
Renca 细胞皮下注射造模注射制备的小鼠肾癌模型对小鼠损伤较小，小鼠死亡率较低，造模成功的标志便于观察。皮下种瘤造模能够模拟肿瘤成瘤及肿瘤生长过程，且不会直接刺激小鼠其它器官，该造模技术目前较为成熟，　且造模成功后的各种指标都能满足研究需要，受到诸多学者的青睐。
河南省中医院动物实验中心具有完备的 IVC 笼盒系统，能够满足实验需求 SPF 级环境。</td></tr>
</table>

对伦理委员有无回避要求：<br>无
声明：<br>1. 我将自觉遵守实验动物福利伦理相关法规和各项规定，同意接受伦理委员会和实验动物室管理者的监督与检查。<br>2 本人保证本申请表中所填内容真实、详尽。<br>声明人：课题负责人签（章）[illegible]<br>动物实验负责人签（章）宋[illegible]<br>年 月 日
实验动物设施意见：<br>设施负责人签（章）河南省中医院（河南中医药大学第二附属医院）中心实验室<br>年 月 日
实验动物福利伦理审查委员会审查意见：<br>审查委员表决：<br>主任委员签（章）河南省中医院（河南中医药大学第二附属医院）伦理委员会<br>年 月 日
备注： □初审； □ 第 次审查

说明：

1. 申报时，请提交本表一式两份及电子版。
2. 受理文号和批准文号由伦理委员会填写。
3. 必须写明所有实验参与人员，不在表中人员动物中心有权拒绝其进入动物房。
4. 此表需双面打印，并签字盖章。

Highlight

1. The vital targeted-molecular-mechanism of Lathyrol against the renal cell carcinoma were interpreted.
2. The anti-tumor effect of Lathyrol was explored via experiments in vivo.
3. The anti-cancer treatment effect of Lathyrol and clinical chemotherapy drugs was compared.
4. The expression of AR, SPHK-2 and related proteins affected via Lathyrol were evaluated.
5. It provides new theoretical options for clinical diagnosis and treatment of renal cell carcinoma

Statement of Non-duplication

This manuscript is a unique submission and is not being considered for publication by any other source in any medium. Further, the manuscript has not been published, in part or in full, in any form.

Statements in Experimental Animals for Researches

The Central Laboratory Ethics Committee of the Second Affiliated Hospital of Henan University of Traditional Chinese Medicine stipulates that the researchers of vivo experiments must comply with the following statements:

1. Experimental animals must be the SPF level or above.
2. During the experiment, the harm to the animal should be minimized, and near-death, death, and severe pain and suffering of the animal should be avoided to the greatest extent possible.
3. In the tumor therapeutic experiment, the tumor load should not exceed 20% of the body weight of the mice. In general experiments, the tumor load should not exceed 10% of the body weight of mice.
4. The diameter of subcutaneous tumor in the back of mice was <25mm, and the diameter of subcutaneous tumor in the back of rats was <40mm.
5. The axillary tumors of the limbs cannot reach the position that seriously affects the normal function of the limbs of mice and rats, or cause pain to the animals due to tumor growth.
6. Animal weight loss should not exceed 20% of normal body weight.
7. No ulceration or infection can occur at tumor growth points.

Statements in Experimental Animals for Researches

The all authors declare that the maximal tumor size/burden was not exceeded relevant guidelines and regulations. The vivo study conforms to follows the above statement and the relevant regulations of the Central Laboratory Ethics Committee of the Second Affiliated Hospital of Henan University of Traditional Chinese Medicine. All methods were carried out in accordance with relevant guidelines and regulations. The study conforms to the ARIVE guidelines and should be carried out in accordance with the 1964 Declaration of Helsinki and its later amendments or comparable ethical standards, the U.K. Animals (Scientific Procedures) Act, 1986 and associated guidelines, EU Directive 2010/63/EU for animal experiments, or the National Institutes of Health guide for the care and use of Laboratory animals (NIH Publications No. 8023, revised 1978).
